# The balance between mitotic death and mitotic slippage in acute leukemia: a new therapeutic window?

**DOI:** 10.1186/s13045-019-0808-4

**Published:** 2019-11-26

**Authors:** Andrea Ghelli Luserna di Rorà, Giovanni Martinelli, Giorgia Simonetti

**Affiliations:** 1 Biosciences Laboratory, Istituto Scientifico Romagnolo per lo Studio e la Cura dei Tumori (IRST) IRCCS, Via P. Maroncelli 40, 47014 Meldola, FC Italy; 2 Istituto Scientifico Romagnolo per lo Studio e la Cura dei Tumori (IRST) IRCCS, Via P. Maroncelli 40, 47014 Meldola, FC Italy

**Keywords:** Acute leukemia, Mitotic death, Mitotic slippage, Mitotic inhibitors

## Abstract

Mitosis is the process whereby an eukaryotic cell divides into two identical copies. Different multiprotein complexes are involved in the fine regulation of cell division, including the mitotic promoting factor and the anaphase promoting complex. Prolonged mitosis can result in cellular division, cell death, or mitotic slippage, the latter leading to a new interphase without cellular division. Mitotic slippage is one of the causes of genomic instability and has an important therapeutic and clinical impact. It has been widely studied in solid tumors but not in hematological malignancies, in particular, in acute leukemia. We review the literature data available on mitotic regulation, alterations in mitotic proteins occurring in acute leukemia, induction of prolonged mitosis and its consequences, focusing in particular on the balance between cell death and mitotic slippage and on its therapeutic potentials. We also present the most recent preclinical and clinical data on the efficacy of second-generation mitotic drugs (CDK1-Cyclin B1, APC/CCDC20, PLK, Aurora kinase inhibitors). Despite the poor clinical activity showed by these drugs as single agents, they offer a potential therapeutic window for synthetic lethal combinations aimed to selectively target leukemic cells at the right time, thus decreasing the risk of mitotic slippage events.

## Background

### Stress during mitosis: cell division, death, or mitotic slippage?

**Fig. 1:**
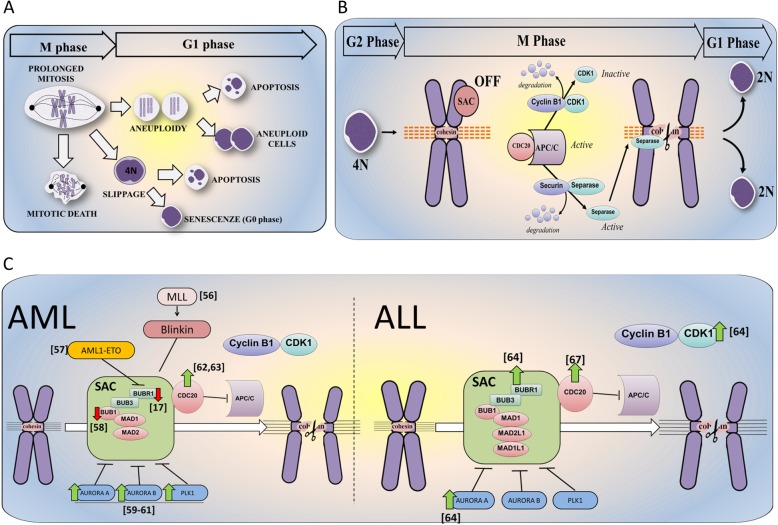
Different cell fate after prolonged mitosis and regulation of mitosis. **a** Graphic representation of the different cell fates after prolonged mitosis. Severe DNA damages or prolonged mitosis can induce direct apoptosis. Mitotically arrested cells can generate aneuploid cells due to premature cytokinesis or chromosome mis-segregation. Aneuploid cells may die during the next cell division or continue to proliferate as viable cells. Cells can overcome the arrest throughout the mitotic slippage generating tetraploid cells. These cells can arrest in interphase and eventually enter cellular senescence or die after slippage [10, 11]. **b** When the kinetochore is properly attached, CDC20 is released and activates APC/C that orchestrates cyclin B1 and securin degradation. Separase in turn degrades the cohesion complex at and near sister chromatid kinetochores promoting mitotic exit [6, 12]. **c** The SAC complex regulates mitosis. Restriction is achieved through inhibition of the activating APC/C-subunit CDC20. Loss of function of SAC proteins such as BUBR1, MAD2, or BUB1 (shown as red arrows) reduces the accuracy of SAC complex and, consequently, promotes genomic instability [13–16]. In parallel, the overexpression of mitotic kinases such as PLK1, Aurora A and B (shown in green arrows) enhances mitotic exit even in the presence of mitotic arrest [17–22]

Cell cycle phases in eukaryotic cells have a precise time duration that varies considerably according to cell type [1]. For example, in a highly proliferating human cell with a total doubling time of 24 h, the G1 phase may last around 11 h, S phase around 8 h, G2 around 4 h, and mitosis around 1 h [2]. Mitosis is the process whereby an eukaryotic cell divides into two identical copies. One of the most important regulators of cell division is the cyclin-dependent kinase 1 (CDK1)-Cyclin B1 complex, also known as mitotic promoting factor (MPF), which controls the progression from G2-phase to M-phase and mitosis itself [3]. CDK1 is a crucial regulator of mitotic transition and its activation drives entry into mitosis [4, 5]. MPF activity must be sustained from prophase to metaphase and subsequently turned off when cells enter anaphase. Then, MPF activity is also needed to exit mitosis [6]. MPF inactivation is obtained by the destruction of cyclin B1 through proteasome degradation and is one of the mechanisms that regulates the precise time duration of mitosis. Several studies have investigated the mechanisms underlying mitotic delay and the biological consequences of so-called “prolonged mitosis.” Different types of stimuli such as oncogene activation, DNA damages, or chromosome mis-segregation/supernumerary chromosomes induce prolonged mitosis [7]. Given that mitosis is physiologically restricted to a precise time frame (minutes), prolonged mitosis (1.5–2.5 h or more) can lead to catastrophic consequences such as aneuploidy and malignant transformation [8] However, it has been reported that in eukaryotic cells, the mitotic checkpoint can hold cells in mitosis for 12–48 h [9]. Mitotically arrested cells display mutually exclusive fates (Fig. 1a): (i) complete cell division after mitotic arrest (frequently accompanied by mis-segregation of chromosomes, thereby leading to aneuploidy); (ii) cell death in mitosis; (iii) a phenomenon known as “checkpoint adaptation” or “mitotic slippage” in which cells return to interphase without cellular division (as tetraploid cells) [10, 11]. These cells can arrest in interphase and eventually enter cellular senescence or die after slippage [11, 23].

### Cell division after mitotic arrest

Mitosis progression is under the control of the spindle assembly checkpoint (SAC), which selectively inhibits the anaphase promoting complex/cyclosome (APC/C) until all the kinetochores are properly attached to microtubules [24]. When the kinetochores are properly attached, the “SAC is satisfied” and SAC proteins (MAD1L1, BUB1, BUBR1, CDC20, BUB3, and MAD2L1) are released [6, 12]. Among them, CDC20, binds and activates APC/C which, in turn, polyubiquitinates structural and regulatory proteins needed for chromatid disjunction and mitosis exit, including securin and cyclin B1 [25, 26] (Fig. 1b). Securin degradation releases separase which cleaves cohesin molecules at and near sister chromatid kinetochores. As a consequence of cohesin cleavage, chromatids segregate in opposite directions at the end of anaphase [13]. Acute myeloid leukemia (AML) cell lines and primary samples display reduced BUBR1 expression compared with normal CD34^+^ bone marrow precursors and acute lymphoblastic leukemia (ALL) blasts [27] (Fig. 1c). Given the role of BUBR1 as an APC/C inhibitor, AML cells with reduced BUBR1 expression are able to progress through mitosis even in the presence of DNA structural aberrations, thanks to APC/C activation, which drives proteasome-dependent degradation of cyclin B1 and securin. Therefore, AML cells can skip mitotic arrest, leading to premature sister chromatid separation [27]. In addition, aberrant securin regulation, causing aneuploidy, has been reported in AML cells carrying *NUP98* translocation [28] (Fig. 1c). The expression of NUP98 fusions causes premature securin degradation in the presence of unsatisfied SAC, by interacting with APC/C^Cdc20^ and displacing BUBR1 [28, 29]. In parallel, degradation of cyclin B1 results in MPF complex inactivation and reversal of the CDK1 phosphorylation cascade by cellular phosphatases (e.g., PP1 and PP2A), which remove CDH1 inhibitory phosphorylation. Moreover, MAD2L2, which binds CDH1 during early mitosis, is degraded at anaphase, thus driving APC/C^CDH1^ activation [30, 31]. CDH1 expression is decreased in AML blasts compared with normal CD34^+^ cells and its downregulation was shown to inhibit cell differentiation in acute promyelocytic leukemia [32]. This evidence suggests that general mechanisms and subtype-specific alterations contribute to the aberrant regulation of cell division in acute leukemia cells.

While non-malignant cells have a remarkably limited tolerance for mitosis duration and delayed mitosis frequently ends in cell death, cancer cells tend to tolerate mitotic delay and the consequences of aberrant mitosis, such as an abnormal chromosome number [33]. Recently, mitotic errors and prolonged mitosis have been linked to chromothripsis, a form of catastrophic chromosomal rearrangement originating from one-step genomic event [34] found in leukemias [35–37] and other tumors [38]. Chromothripsis originates from genomic fragility of micronuclei containing lagging chromosomes. Micronuclei genomic instability is a consequence of nuclear envelope collapse, which occurs during interphase and hampers the capacity of sensing and repairing DNA damage [39]. It has been shown that lagging chromosomes undergo aberrant nuclear envelope assembly, with regular involvement of “core” nuclear envelope proteins, in the absence of nuclear pore complexes and other “non-core” nuclear envelope proteins [40]. The recruitment of nuclear envelope proteins is partly inhibited by Aurora B kinase through regulation of PLK1 activity, which needs to be switched off for loading of nuclear pore complexes at lagging chromosomes [41]. Moreover, a major mechanism underlying chromothripsis is represented by the inhibitory function of spindle microtubules on proper nuclear envelope assembly that in turn results in the lack of key proteins required for preserving genomic integrity in micronuclei [39, 40]. Therefore, prolonged mitosis, by disrupting spindle microtubules and mitotic exit dynamics, can trigger genomic catastrophic events.

### Cell death in mitosis

**Fig. 2:**
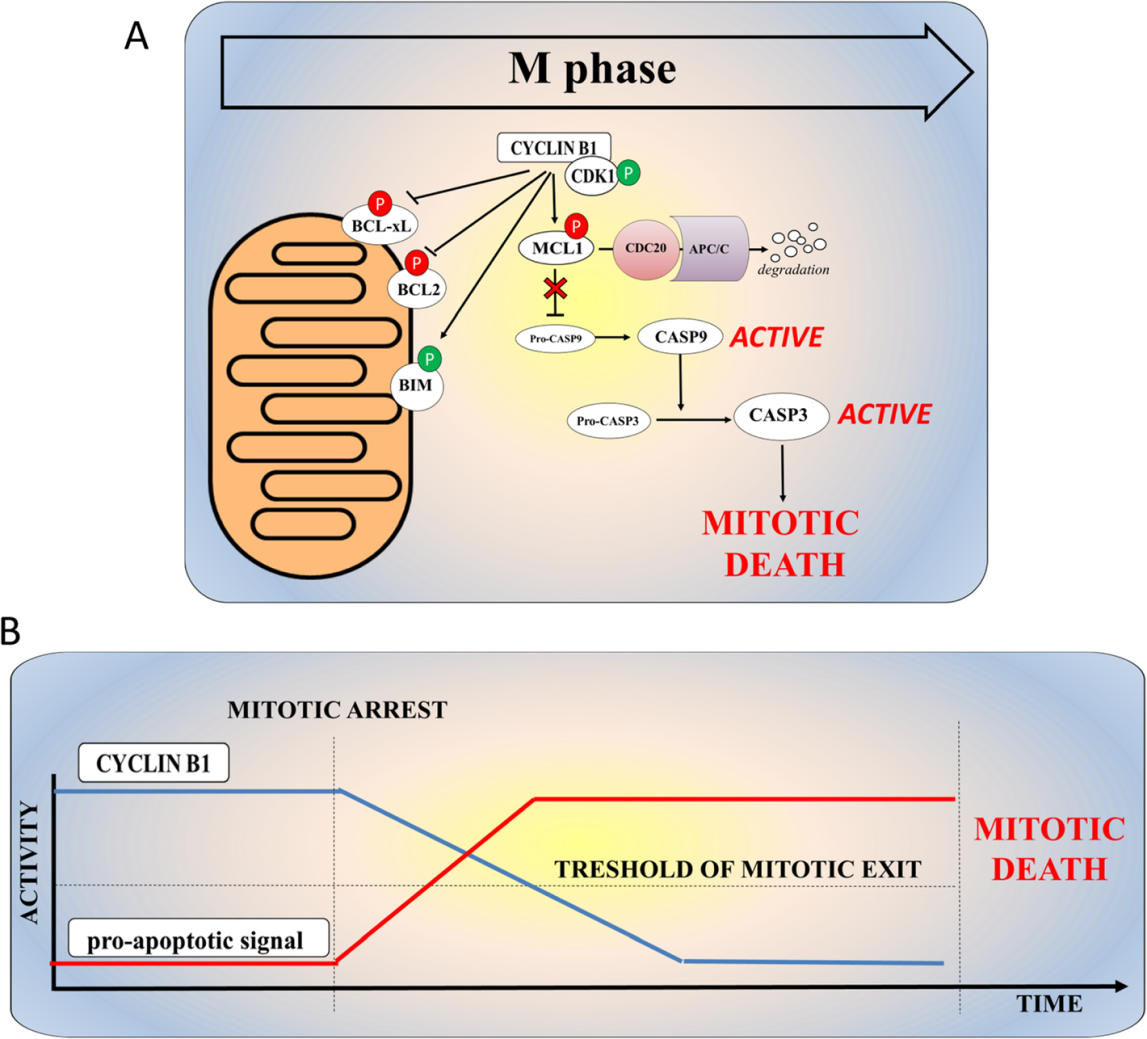
Mechanism of cell death during mitotic arrest. **a** MPF complex promotes the induction of apoptosis through direct phosphorylation of different pro-/anti-apoptotic factors. CDK1-mediated inactivating phosphorylation of the BCL2 and BCL-xL and activatory phosphorylation of the pro-apoptotic BIM. MPF complex also promotes the degradation of MCL-1 via APC/CCDC20. MCL1 loss promotes caspase activation leading to cell death [43–45]. **b** Graphic representation of the timing of reduction of Cyclin B1 level and the increment of pro-apoptotic signals in the contest of mitotic cell death. Rapid induction of pro-apoptotic signals in the presence of active MPF complex induces apoptosis during prolonged mitosis

Cell death in mitosis is an onco-suppressive mechanism that targets cells experiencing defective mitoses in order to preserve genetic integrity [42] and several molecular players are involved in its regulation (Fig. 2a and b). First, the timed degradation of the apoptosis inhibitory proteins, including BCL2, BCL-xL, and MCL1, can activate the apoptotic response and the MPF complex balances pro-apoptotic and anti-apoptotic signals. In AML cells, the exposure to microtubule poison, vinblastine, increases the amount of Cyclin B1 and the inhibitory phosphorylation of BCL-xL (Ser62), leading to the cleavage of PARP proteins and cell death [46]. Rapid induction of pro-apoptotic signals in the presence of active MPF complex also induced apoptosis during prolonged mitosis. For example, the pro-apoptotic BH3-only family member, BIM, undergoes CDK1-dependent phosphorylation in leukemia K562 cells following treatment with the microtubule targeting agents. This phosphorylation of BIM at the mitochondria correlates with mitotic arrest and precedes cell death [43]. Moreover, prolonged mitosis induced by inhibition of sphingosine kinase 1 results in CDK1-mediated inactivating phosphorylation of the pro-survival proteins BCL-2 and BCL-xL and degradation of MCL1, ultimately leading to apoptosis [44]. In addition to pro-apoptotic and anti-apoptotic protein regulation, the induction apoptosis in mitotically arrested cells has been associated with the accumulation of DNA damages during prolonged mitosis. Several studies showed that mitotic arrest is an intrinsic stimulus leading to the activation of the DNA damage response (DDR) [47, 48]. Mitotically arrested cells display a massive induction of γH2AX foci, which are markers of DNA damage sites, especially on telomeres [49]. Telomere-specific damages are enhanced by treatment with either the MCL1/BCL2/BCL-xL inhibitor, Obatoclax and is caused by the endonuclease activity of the caspase-activated DNase (CAD), which is released from its inhibitor by caspase 3 activation at telomere regions [50]. This lead to ATM and DNA-PK activation which in turn activates a P53-dependent response [8, 48]. In unperturbed cells, the tumor suppressor protein P53 is phosphorylated, stabilized, and activated during mitosis and, more precisely, during mitotic exit [51]. Activation of the P53 pathway during mitotic arrest has been extensively studied to understand how cancer cells respond to mitotic poisons (spindle or topoisomerase inhibitors). The main consequences of P53 activation are cell cycle arrest (G1 checkpoint), induction of DNA damage repair, and activation of apoptosis. Prolonged mitotic arrest induced by mitotic drugs (i.e., microtubule poisons) causes apoptosis in responsive cell lines, while cells with low sensitivity undergo slippage into tetraploid G1 cells, followed by P53-dependent arrest or apoptosis of daughter cells [48]. The events leading from prolonged mitosis to P53 activation have been organized into three hypothetical models: (i) according to the “mitotic clock” model, cells should sense the duration of mitosis and slowly accumulate P53 during mitosis [21, 52]; (ii) in the “DNA ploidy or centrosome counter checkpoint” model, P53 activation should occur in tetraploid cells after mitotic slippage or cytokinesis inhibition. Orth and colleagues demonstrated that P53 is not induced in apoptosis-sensitive cell lines that die during mitotic arrest, likely due to physiological inhibition of transcription and translation during mitosis [48]. However, in apoptosis-resistant cell lines, DNA damage mediates post-slippage P53 induction [50], which is induced by Aurora kinase A and B [53]. This event prevents cells from entering the cell cycle through the upregulation of the CDK inhibitor P21 [48]. We have recently showed that aneuploid AML cells have a transcriptional signature of P53 pathway inactivation, which occurs on a structural or functional base [54] and, in particular, chromothripsis [35] associates with *TP53* genomic lesions. In this scenario, inactivation of p53-dependent apoptosis may result in the survival and proliferation of AML clones with complex genomic abnormalities.

### Mitotic slippage

**Fig. 3:**
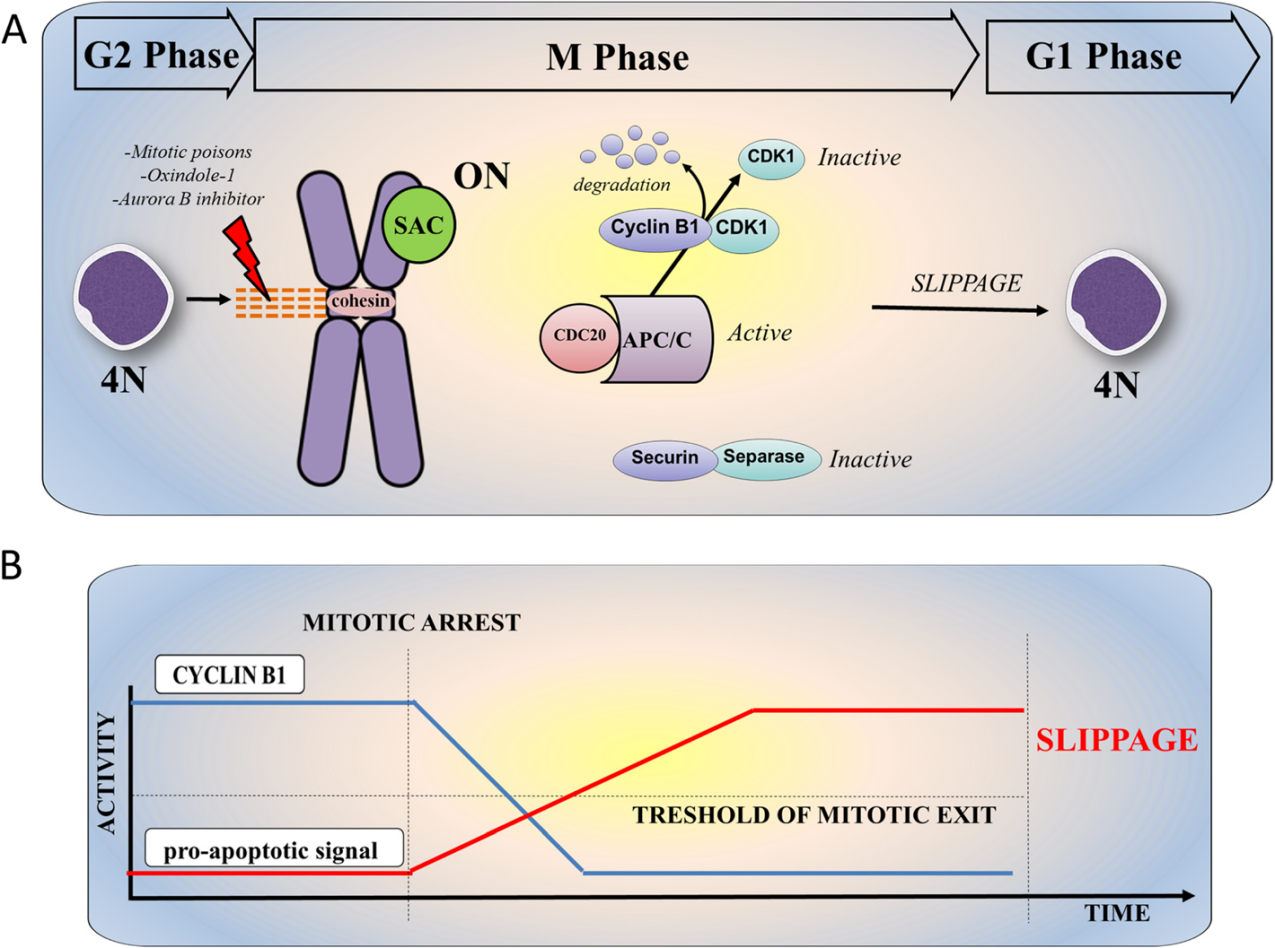
Mechanism of cell slippage during mitotic arrest. **a** Rapid degradation of Cyclin B1 and slow induction of pro-apoptotic signals (or slow degradation of pro-survival proteins) during mitotic arrest caused by mitotic poisons or Aurora B inhibition can lead to slippage [56]. During the mitotic slippage, APC/CCDC20 degrades Cyclin B1 but not securin. The reduction of Cyclin B1 level below mitotic exit threshold induced mitotic exit without cell division. **b** Graphic representation of the timing of reduction of Cyclin B1 level and the increment of pro-apoptotic signals in the contest of mitotic slippage. In the presence of active MPF complex, the rapid degradation of Cyclin B1 level below the mitotic exit threshold induces mitotic slippage during prolonged mitosis

Mitotic slippage allows cell escape from death [7]. Several studies have shown that treatment failure using microtubule poisons such as taxanes (e.s. paclitaxel) and Vinca alkaloids (e.s. vinblastine and vincristine) is due to mitotic slippage in cancer cells [10, 52, 55] (Fig. 3a and b). From a mechanistic point of view, rapid degradation of Cyclin B1 and slow induction of pro-apoptotic signals (or slow degradation of pro-survival proteins) during mitotic arrest can lead to slippage (e.g. generation of polyploid cells) [56]. Evidence suggests that overexpression of the anti-apoptotic protein BCL2 can prevent death of mitotically arrested cells long enough for Cyclin B1 levels to drop below a threshold for mitotic slippage [7]. Conversely, rapid induction of pro-apoptotic signals in the presence of active Cyclin B1 usually leads to cell death [10].

In AML cells, the relationship between Cyclin B1 degradation and the induction of mitotic slippage has been confirmed using Oxindole-1 (OX-1) [46]. Indeed, OX-1 treatment promotes the gradual degradation of Cyclin B1, even if the SAC is not satisfied. Cyclin B1 degradation in turn compromises the ability of MPF complex to promote apoptosis (loss of BCL-xL phosphorylation), thus generating viable polyploid cells through mitotic slippage. This phenomenon was reversed by combining OX-1 treatment and BCL2 family inhibitors that forced AML cell apoptosis [46]. Moreover, inhibition of the CDK1 kinase activity favors mitotic slippage, polyploidy, and resistance to apoptosis in AML cells through BCL-xL stabilization, which is reversed by combined suppression of BCL-xL by ABT-263 [46]. Inhibition of Aurora B (but not Aurora A) also promotes mitotic slippage in P53-proficient cells, resulting in post-slippage P53 activation which, in turn, leads to cell cycle arrest and apoptosis [53]. In apoptosis-resistant cell lines, post-slippage P53 induction [50], which is induced by Aurora kinase A and B [53], prevents cells from entering the cell cycle through the upregulation of the CDK inhibitor P21 [48]. Despite the initial enthusiasm in Aurora kinase targeting (addressed later in the review), which was supported by their overexpression in AML cells, some concerns have been raised, due to the induction of polyploidy [57] which can occur in the absence of P53-mediated surveillance. MCL1 levels also influence slippage, with MCL1 overexpression expanding the time from mitotic entry to mitotic exit in the presence of taxol, while its inhibition leads to acceleration [56]. In this scenario, MCL1 competes with Cyclin B1 for binding to components of the proteolysis machinery, thereby slowing down the degradation of Cyclin B1 [56]. Taken together, evidence highlights the need of a timely and balanced regulation of the cell cycle machinery and the apoptotic signals during mitotic arrest, which determines the slippage versus death cellular fate. The intrinsic APC/C-mediated regulation and proteasome-dependent degradation of several pro-apoptotic and anti-apoptotic proteins play a crucial role in the fine-tuning of the cellular response and suggest that the combination of drugs targeting anti-apoptotic proteins and mitotic players can provide synergistic effects. Accordingly, it has been recently shown that the TP53 apoptotic network, and in particular inactivation of TP53, BAX, and PMAIP1, mediates resistance to BCL2 inhibition in AML [58]. A deep understanding of the precise timing of activation of mitotic players and post-translational modifications of anti-apoptotic proteins may improve the selection of the most effective combinations to kill cells during mitotic arrest, cell division, or slippage.

### Molecular alterations targeting late mitosis and its regulation in acute leukemia

Prolonged mitosis is a common event in human cancer [59]. There is evidence that structural or functional inactivation of tumor suppressor genes can induce mitotic arrest. Inactivation of retinoblastoma (*RB*) through deletion or mutations, which is a relatively rare event in both AML [60] and ALL [61–63], induces constitutive or temporary MAD2 overexpression which, in turn, delays mitosis and drives genetic instability and aneuploidy [64, 65]. Given that SAC controls progression through mitosis by regulating APC/C function and that its defective activity can lead to errors in chromosome segregation and division of cells carrying DNA damage, this surveillance mechanism is considered a major candidate for genomic alterations in human cancer, especially those exhibiting high levels of genomic instability (e.g., aneuploid tumors) [66]. However, SAC proteins are rarely mutated in solid tumors or in leukemia [54]. Conversely, SAC attenuation and deregulated expression of its components occur in several cancer types and also in acute leukemia cells (Fig. 1c). Failure of the SAC complex to sustain prolonged mitosis in response to mitotic inhibitors is responsible for the low sensitivity of AML cells to spindle poisons, which can be restored by BUBR1 or cyclin B1 induction. Compared with AML cells, lymphoblastic cell lines show higher BUBR1 expression and its knockdown reduces the accumulation of G2/M cells in response to anti-mitotic drugs [27]. Along with deregulated BUBR1, a leukemogenic MLL-fusion protein involving the mitotic regulator Blinkin (alias AF15q14) was shown to interfere with SAC activity [14]. Indeed, Blinkin participates to the recruitment of BUBR1 and BUB1 to the kinetochores and regulates their attachment to spindle microtubules, thus suggesting a causative role in genomic instability in this subgroup of MLL-driven leukemia. Moreover, AML cells expressing a C-terminally truncated form of the AML1/ETO fusion protein display attenuation leading to aberrant chromosome segregation and aneuploidy [15]. Mitotic arrest following nocodazole treatment is reduced in these cells, due to downregulation of mitotic checkpoint proteins including BUBR1 and mainly securing.

Deregulated SAC components and mitotic kinases contribute to defective mitosis in AML, with BUB1 downregulation promoting mitotic checkpoint weakening [16]. Leukemic cells also overexpress Aurora A, Aurora B, and PLK1, which induce mitotic checkpoint failure, chromosome mis-segregation, and aberrant cell cycle control, respectively [17–19]. Moreover, we and others recently showed that aneuploid and complex karyotype AML exhibits higher levels of PLK1 and CDC20 expression than euploid AML, suggesting their potential usefulness in synthetic lethal therapeutic combinations [20, 21].

The scenario is slightly different in ALL, a field in which few studies have been conducted. BUBR1, CDK1, and Aurora kinase A [22] have been shown to be upregulated in relapsed childhood B-ALL cases with respect to matched leukemic samples at diagnosis [67]. *Oliveira* and colleagues reported that PLK1 expression was heterogeneous in pediatric ALL cases, with no differences between the overall population and normal bone marrow cells [68]. Moreover, genes involved in spindle organization (*SPC25*, *KIF11*, *ESPL1*, *UBE2C*, *TACC3*), chromosome segregation (*SPC25*, *NUSAP1*, *BIRC5*, *CENPE*, *ESPL1*, *PTTG1*), and cell division (KIF11, *CCNF*, *NUSAP1*, *CENPE*, *CDC20*) have been found to be upregulated in pediatric B cell precursor ALL compared with normal B cell progenitors, highlighting their potential for use as therapeutic targets [69]. With regard to adult ALL cases, mRNA levels of two negative regulators of CDK1-cyclin B1 complex, WEE1 and PKMYT1, are significantly upregulated in leukemic samples with respect to normal hematopoietic precursors [70] .

### Preclinical and clinical data of compounds targeting mitotic stability in acute leukemia

In the last few years, several molecules have been developed that selectively target the mitotic apparatus to prevent cell duplication or enhance cell death. Here we summarize the preclinical and clinical data available in AML and ALL on the use of microtubule targeting agents (MTA) and second-generation mitotic drugs, including “mitotic blockers,” that delay mitosis progression and exit, and “mitotic drivers,” that force mitotic exit. Table 1 shows the clinical trials on targeted mitotic inhibitors used as single agents or in combination.

**Table 1:** Clinical trials of targeted mitotic inhibitors

Target	Study	Patients^a^	Phase	Status^b^	ClinicalTrials.gov identifier
CDK2, CDK5, CDK1, CDK9	Venetoclax and dinaciclib (MK7965)	AML, relapsed/refractory	I	Recruiting	NCT03484520
CDK2, CDK5, CDK1, CDK9	Dinaciclib or gemtuzumab ozogamicin	AML/ALL, relapsed/refractory	II	Terminated	NCT00798213 [71]
PLK1 and other kinases	Oral rigosertib	AML, ALL, MDS, CLL, CML, relapsed/refractory	I	Completed	NCT00854646 [72]
PLK1 and other kinases	Rigosertib	AML, ALL, CML, relapsed/refractory, transformed MPN	I/II	Completed	NCT01167166 [73]
PLK1 and other kinases	Oral rigosertib	AML, MDS, relapsed/refractory or ineligible for standard chemotherapy	I/II	Completed	NCT00854945 [74]
PLK1 and other kinases	Oral rigosertib in combination with azacitidine	MDS, CMML, RAEB-t/non-proliferative AML	I/II	Active, not recruiting	NCT01926587 [75]
PLK1	Volasertib	AML, relapsed/refractory or ineligible for standard induction therapy	I	Completed	NCT01662505 [76]
PLK1	Volasertib	Pediatric leukemia, relapsed/refractory, advanced solid tumors, no available effective treatments	I	Completed	NCT01971476 [77]
PLK1	BI 6727 (volasertib) as monotherapy or in combination with cytarabine	AML, relapsed/refractory or ineligible for intensive induction therapy	II	Active, not recruiting	NCT00804856 [78, 79]
PLK1	Volasertib in combination with low-dose Cytarabine	Newly diagnosed AML, aged ≥ 65 years, ineligible for intensive induction therapy	III	Active, not recruiting	NCT01721876 [80]
PLK1	Volasertib in combination with decitabine	AML, aged ≥ 65 years, newly diagnosed and ineligible for standard intensive therapy or relapsed/refractory	I	Terminated	NCT02003573
PLK1	Intensive chemotherapy with or without volasertib	AML, newly-diagnosed, high-risk MDS	II	Terminated	NCT02198482
PLK1	Onvansertib in combination with either low-dose cytarabine or decitabine	AML, relapsed/refractory or ineligible for intensive induction therapy	Ib/II	Recruiting	NCT03303339 [81]
PLK4 (and Aurora B)	CFI-400945	AML, MDS, relapsed/refractory	I	Recruiting	NCT03187288
Aurora A	MLN8237	Recurrent childhood AML, ALL, solid tumors	II	Completed	NCT01154816 [82]
Aurora A	MLN8237	AMKL, MF	I	Active, not recruiting	NCT02530619 [83]
Aurora A	MLN8237	AML, relapsed/refractory or ineligible for intensive induction therapy, high-grade MDS	II	Completed	NCT00830518 [84]
Aurora A	MLN8237 in combination with 7+3 induction chemotherapy	Newly diagnosed AML	I	Completed	NCT01779843 [85]
Aurora A	MLN8237 with induction chemotherapy	High-risk AML, newly diagnosed	II	Active, not recruiting	NCT02560025 [86]
Aurora A	MLN8237 in combination with vorinostat	B/T ALL, CLL, lymphoma, relapsed/refractory	I	Completed	NCT01567709 [82]
Aurora B	AZD1152	AML, relapsed/refractory or ineligible for other treatments	I	Completed	NCT00530699 [87]
Aurora B	AZD1152	AML, relapsed/refractory or ineligible for other treatments	I/II	Completed	NCT00497991 [88]
Aurora B	AZD1152 in combination with low-dose cytosine arabinoside (LDAC)	AML, newly diagnosed, ineligible for other treatments, aged ≥ 60	I	Completed	NCT00926731 [89]
Aurora B	AZD1152 alone and in combination with low dose cytosine arabinoside (LDAC)	AML, newly diagnosed, aged ≥ 60	II/III	Completed	NCT00952588 [90]
Aurora B	AZD2811 nanoparticles alone and in combination with azacitidine	AML, high-risk MDS	I/II	Recruiting	NCT03217838 [91]
Aurora A/B and other kinases	AT9283	Acute leukemia, childhood, adult, relapsed/refractory,	I	Completed	NCT01431664 [92]
Aurora A/B and other kinases	AT9283	AML, ALL, relapsed/refractory or ineligible for standard therapy, high-risk MDS, refractory CML	I/II	Terminated	NCT00522990 [93]
Aurora A/B	Orally administered AMG 900	AML, refractory or ineligible for standard therapies	I	Completed	NCT01380756 [94]
Aurora A/B and other kinases	MK-0457	CML, Ph+ ALL	I	Terminated	NCT00500006 [95]
Aurora A/B and other kinases	MK-0457	T315I mutant CML, Ph + All	II	Terminated	NCT00405054 [96]
Aurora A/B and other kinases	MK-0457	AML, relapsed/refractory, high-risk MDS, B-ALL, myeloproliferative diseases, CML in blast crisis	I/II	Completed	NCT00111683 [97]
Aurora A/B and other kinases	AS703569/MSC1992371A	AML, ALL, CLL, NHL, relapsed/refractory, or ineligible for standard therapy; high-risk MDS, CML, resistant or intolerant to standard treatment; myeloproliferative disorders with no effective treatment options.	I	Terminated	NCT01080664 [98]
Aurora A and other kinases	ENMD-2076	AML, ALL, CLL, relapsed/refractory; high-risk MDS, CML	I	Completed	NCT00904787 [99]

*ALL* acute lymphoblastic leukemia, *AMKL* acute megakaryoblastic leukemia, *AML* acute myeloid leukemia, *CLL* chronic lymphocytic leukemia, *CML* chronic myeloid leukemia, *CMML* chronic myelomonocytic leukemia, *MDS* myelodysplastic syndrome, *MF* myelofibrosis, *na* not applicable, *Ph* philadelphia chromosome

^a^Adult patients if not specified

^b^Withdrawn studies were not reported

### Conventional mitotic inhibitors

#### Microtubule poisons

Microtubule targeting agents (MTAs) such as vinca alkaloids, which suppress microtubule dynamics in the mitotic spindle to activate the SAC, are widely used in the clinical treatment of different types of oncohematological malignancies. Although the mechanisms of action of these drugs are only partially known, prolonged mitotic arrest appears to be a major player in the anti-proliferative activity of mitotic poisons. Vincristine (VCR), an anti-tubulin compound extracted from *Catharanthus roseus*, is frequently used in combination with chemotherapy for the treatment of different cancer types [100, 101]. Recently, *Kothari* and colleagues identified a potential mechanism of action of VCR in ALL, hypothesizing that the effect was dependent on the cell cycle phase in which the cell interacts with the anti-tubulin compound. Primary ALL cells in G1 phase were directly induced to cell death, while cells that had passed the so-called “microtubule sensitivity checkpoint” and were in late G1/early S phase continued to cycle. These cells underwent cell cycle arrest and cell death during mitosis [102] . Vinblastine is another vinca alkaloid that binds to the ends of microtubules, suppresses their dynamic instability, and causes their depolymerization [103] . Different studies on leukemia and lymphoma have shown a variable response to vinblastine. One of the reported mechanisms of action of vinblastine, which correlates with sensitivity, is related to the expression of the anti-apoptotic marker MCL1. Vinblastine induces MCL1 expression via ERK1 signaling activation already after 2-h treatment in the ML-1 AML cell line [104], as a protective mechanism to acute apoptosis. Indeed, MCL1 suppression sensitized selected cell lines to vinblastine treatment. This evidence further supports the therapeutic potential of drug combinations acting on mitosis and apoptosis, and indicates that the selection of the right anti-apoptotic target (MCL2, BCL2, BCL-xL), according to the mitotic phase and patient-specific dependencies, is crucial to maximize treatment efficacy.

Microtubule-stabilizing agents (MSAs) are a second class of mitotic poisons. Among them, Paclitaxel (PTX) is the most used for the treatment of different kind of tumors. A PTX induces cell death mainly by preventing microtubule depolymerization and, consequently, inducing SAC complex inhibition of cell cycle progression (arrest in G2/M phase) [105, 106]. In acute leukemia cells, the effect of PTX on the regulation of mitosis has not been investigated yet.

### Second-generation mitotic drugs

#### CDK1-cyclin B1 inhibitors

Hundreds of studies have shown the efficacy of different pan-/multi-CDK inhibitors in hematological diseases [107, 108]. Few data have been reported on the activity of Seliciclib (Roscovite), a CDK1, 2, 5,7, 8, and 9 inhibitor, in acute leukemia. In particular, *Qi* and colleagues observed a dose-dependent response in AML models [108]. Conversely, there is evidence of a growth inhibitory and anti-neoplastic effect of the CDK1, 2, 5, and 9 inhibitor dinaciclib (SCH 727965) on AML and T-ALL cell lines in vitro and in vivo [44, 45]. T-ALL cells treated with dinaciclib showed decreased expression of several pro-survival proteins including survivin, cyclin T1, and c-MYC. The treatment also increased the number of cells in G2/M phase and induced apoptosis via the intrinsic pathway, by downregulating MCL-1 [45, 71]. In a phase II trial (NCT00798213 [71]) conducted in adult patients with advanced AML (≥ 60 years) or ALL, dinaciclib demonstrated anti-leukemia activity in 60% of cases. However, no objective responses were reported and MCL-1 levels rapidly recovered after drug-induced decline [71]. The drug is currently under clinical investigation in combination with the BCL2 inhibitor venetoclax (NCT03484520). The first specific CDK1 inhibitor to be developed is RO-3306, which promotes mitotic arrest in nocodazole-treated cells (G2/M arrest) [109]. RO-3306 impaired protein translation and enforced the expression of origin licensing and replication factors in myeloid NB4 cell line, suggesting cell preparation for genome re-replication [110]. In combination with the MDM2 inhibitor Nutlin-3, RO-3306 enhanced p53-mediated BAX conformational changes and apoptosis in AML cell lines and primary cells [111]. Moreover, RO-3306 cooperated with Nutlin-3 in suppressing BCL-2, P21, and survivin expression, thus inducing apoptosis [111].

#### APC/C^CDC20^ inhibitors

Due to the potential tumorigenic role of CDC20 overexpression and APC/C^CDC20^ hyperactivity across cancers, several inhibitors have been tested (extensively reviewed by *Wang* et al. [112]). However, few studies have been conducted on acute leukemia models. Withaferin A (WA) was shown to reduce cell viability and induce apoptosis and cell cycle arrest at G2/M checkpoint in different acute leukemia cell lines [113, 114] and in primary AML cells [115]. Its anti-tumor activity is related to the inhibition of the transcription factor C/EBPβ [116].WA was also highly effective on AF4/MLL-positive ALL cell lines, inducing cell cycle arrest, activation of the p38-downstream pathway, and DNA damage [117]. Moreover, in T-ALL cell lines, WA synergized with γ-secretase inhibitors (NOTCH inhibitor), reducing cell viability and inducing apoptosis [118].

#### Polo-like kinase inhibitors

Several studies have shown the efficacy of PLK1 suppression by small interfering RNA or selective PLK1 small molecule inhibitors in acute leukemia cells [119]. Among them, rigosertib and volasertib are the most widely studied in acute leukemia. Rigosertib is a non-adenosine triphosphate (ATP) competitive benzyl-styryl-sulfone analog that inhibits PLK1 and other kinases, including PI3K. In a phase I/II study on myelodysplastic syndrome (MDS) and AML patients with relapsed/refractory disease, 53% of cases demonstrated bone marrow/peripheral blood responses or stable disease, with a median survival of 15.7 and 2.0 months for responders and non-responders, respectively (NCT00854646). Volasertib (BI 6727) is an ATP-competitive kinase inhibitor that targets the ATP-binding pocket of PLK1, PLK2, and PLK3. Volasertib has proven effective as monotherapy in leukemia cell lines, primary cells, and xenograft models (especially in AML models) [119, 120], synergizing with different compounds including the proteasome inhibitor (bortezomib) [121], PI3K inhibitor [122], and bromodomain and extra-terminal motif (BET) protein inhibitor (BI 894999) [123]. In a multicenter phase I/II trial (NCT00804856), volasertib was tested as monotherapy and in combination with low-dose cytarabine in patients with relapsed/refractory AML. A higher number of patients receiving the combination obtained complete response (CR) or CR with incomplete blood count recovery (CRi), compared with those receiving cytarabine alone (31.0 vs.13.3%) [78, 79]. Following these promising results, a phase III trial was designed to test volasertib in combination with low-dose cytarabine in previously untreated elderly patients, which were ineligible for intensive chemotherapy (NCT01721876). However, the trial did not meet the primary endpoint and a negative overall survival (OS) trend was reported, due to the high frequency of fatal infections in patients receiving the drug combination [80]. An additional phase I trial of volasertib in combination with decitabine was prematurely terminated (NCT02003573), following the discontinuation of volasertib clinical development. None of the patients completed the treatment and infections were still recurrent (e.g., pneumonia reported in 46% of cases). A phase I dose-escalation study was completed in pediatric patients affected by relapsed/refractory leukemia or advanced solid tumors, with limited anti-tumor and anti-leukemia activity [77]. Recent data from our group and others, showed that PLK1 is highly expressed in aneuploid AML [20], and that complex karyotype AML, including *TP53*-mutated cases, is highly sensitive to volasertib, both in vitro and in vivo, in xenograft models [21]. These insights may open a new and specific therapeutic window for volasertib-based therapeutic combinations. Onvansertib (PCM-075, formerly NMS-1286937) is a highly selective, orally available, ATP-competitive inhibitor of PLK1. It causes G2/M cell cycle arrest followed by apoptosis in AML [124], ALL cell lines and primary cells from pediatric ALL expressing high PLK1 levels [125]. Moreover, it inhibited tumor growth in xenograft models from normal karyotype AML and aggressive CD56^+^ monoblastic AML [126], alone or in combination with cytarabine [124]. Onvansertib also synergized with FLT3 inhibitors [127]. The first results of the phase 1b/2 trial of onvansertib in combination with standard of care have been recently released, showing the greatest anti-leukemic activity in the onvansertib+decitabine arm, with 50% of patients achieving CR (NCT03303339). Regarding PLK inhibitors, a phase I trial with **CFI-400945** is currently ongoing in relapsed/refractory AML and MDS (NCT03187288). CFI-400945 was initially developed to target PLK4 that regulates centrosome duplication and genome integrity and its inhibition showed promising results in preclinical studies of solid tumors [128]. Recently, a dual activity of the drug on both PLK4 and Aurora B has been suggested [129], which may provide an intrinsic synthetic lethal combination in selected models.

#### Aurora kinase inhibitors

Several Aurora kinase inhibitors have progressed through preclinical testing and into phase I or phase II trials, including Aurora A (MLN8237, MK-5108, ENMD-2076 [130], the latter also targeting other kinases), Aurora B (AZD-1152 and AZD-2811), and a number of dual or pan-Aurora inhibitors (AS703569/MSC1992371A [131], MK-0457, AT9283, GSK1070916 [132], AMG 900 [133], PHA-739358). However, few drugs reached advanced clinical phase development in acute leukemias, due to low activity and/or intolerability of a significant patient proportion to the required dose. The efficacy of **MK-5108**, a selective Aurora A inhibitor, in acute leukemia, was investigated in one single study showing cytotoxic and cytostatic activity in T-ALL cell lines and primary cells [134]. The reduction in cell viability was related to a significant activation of G2/M cell cycle checkpoint followed by the induction of apoptosis. The other selective Aurora A inhibitor, MLN8237 (Alisertib), was largely studied in acute leukemia and proved in vitro efficacy, in particular on AML cells [135]. *Kelly* and colleagues showed that MLN8237 used as monotherapy decreased cell viability and colony-forming ability and increased apoptosis in AML models both in vitro and in vivo. The inhibitor also enhanced the cytotoxicity of cytarabine in AML cell lines and primary samples [136]. In the clinical setting, MLN8237 displayed good safety profiles when administered as single agent or in combination with chemotherapy. A phase II clinical trial reported a 13% overall response rate using MLN8237 monotherapy in relapsed/refractory AML and high-risk MDS (NCT00830518 [137]). In the same trial, 17% of AML patients obtained a partial response and 49% had a stable disease. However, a phase II trial of MLN8237 in children and adolescents with relapsed/refractory solid tumors or acute leukemia (AML and ALL) achieved an objective response rate lower than 5% [138]. Similarly, a phase I/II trial of the multikinase inhibitor **AT9283** in children with relapsed or refractory acute leukemia carried out by *Vormoor* and colleagues revealed tolerable toxicity while lacking evidence of efficacy at the explored doses (NCT01431664 [92]). More promising results were obtained by MLN8237 combinatorial approaches. A phase I study (NCT01779843) of MLN8237 in combination with 7+3 induction chemotherapy in patients with AML reported an 86% remission rate (CR + CRi) [85]. Of note, 88% of patients over 65 years of age and 100% of high-risk cases (secondary acute leukemia or cytogenetically high-risk disease) obtained a composite remission. Preliminary results of the phase II trial of MLN8237 in combination with chemotherapy in high-risk patients (NCT02560025) showed a CR + CRi rate of 59% in secondary AML, 67% in patients aged ≥ 65, 77% in adverse risk karyotype, and of 75% *TP53*-mutated cases [86]. The outcome in terms of efficacy, remission rate, and survival (12% of 12-month OS, with a median follow-up of 14 months) is expected to provide positive results, which would represent a strong achievement for such a poor outcome population. The Aurora B inhibitor **AZD1152 (Barasertib)** has been extensively studied in acute leukemias. AZD1152 treatment reduced AML cell proliferation, induced cell death in vitro, and decreased cell growth in xenograft models [139]. These results were replicated in additional preclinical studies that showed inhibition of proliferation and induction of apoptosis in ALL and AML cells in response to Aurora B suppression by AZD1152 [57, 140, 141]. AZD1152 has also been tested in several phase I/II clinical trials, with heterogeneous effectiveness. An overall hematologic response rate of 25% was reached in newly diagnosed or relapsed AML, with manageable toxicities (NCT00497991 [88]) and in particular low neurotoxicity, which was a major issue with MTA. Forty-five percent of overall response rate resulted from a phase I study of AZD1152 with low-dose cytarabine in the elderly (NCT00926731), and a significantly improved objective CR rate was obtained in a phase II study of AZD1152 treatment compared with low-dose cytarabine in the same population (35.4% vs 11.5%, NCT00952588). AZD1152 induced responses across all cytogenetic risk groups, including those with adverse cytogenetic profiles. However, no significant improvement in OS was observed in the phase II study and AZD1152 was discontinued. Recently, *Floc’h* and colleagues studied the efficacy of AZD1152-hQPA (AZD2811), a nanoparticle formulation of AZD1152, in preclinical models of AML. The authors demonstrated that AZD2811 strongly inhibited tumor growth, exceeding the activity of AZD1152. The improved antitumor activity was also associated with increased phospho-histone H3 inhibition, induction of polyploidy, and apoptosis [142]. AZD2811 is currently under clinical investigation as monotherapy and in combination with azacitidine (NCT03217838) and preliminary results showed a good safety profile [91]. Danusertib (PHA-739358) is a potent pan Aurora kinase inhibitor (Aurora-A IC50 = 13 nM, Aurora-B IC50 = 79 nM, Aurora-C IC50 = 61 nM) that also showed inhibitory activity against others kinases, including ABL, RET, and TRK-A. *He* and colleagues reported that danusertib negatively regulates AURKB/p70S6K/RPL15 axis with the involvement of PI3K/Akt/mTOR, AMPK, and p38 MAPK signaling pathways, leading to the induction of apoptosis and autophagy in AML cell lines [143]. Of note, danusertib inhibits both wild-type and T315I-mutant ABL kinase isoforms [144]. Danusertib efficacy was assessed in a phase I study of adult patients with CML in accelerated or blastic phase or with BCR-ABL1-positive ALL resistant or intolerant to imatinib and/or other second generation ABL kinase inhibitors. Four (20%) of the 20 evaluable patients who responded to treatment carried the T315I mutation (NCT00335868) [145].

### Future prospective: drug-to-drug synthetic lethality to target mitotic slippage

Microtubule-binding agents, such as vinca alkaloids or taxanes, are commonly used in the clinics to treat multiple types of cancer but negative side effects, as neuro-/nefro-toxicity and drug resistance, frequently related to the need of a functional SAC for their proper activity, limit their therapeutic applicability [61]. Moreover, several cases of secondary myeloid neoplasia (MDS or AML) were reported after treatment of primary tumors with mitotic poisons such as paclitaxel. The reported cases included different solid tumors carrying a high number of cytogenetic abnormalities and with a very poor prognosis [146–149]. Besides the potential long-term side effects of the drugs, this evidence clearly points to a link between prolonged mitosis, malignant transformation, and the development of leukemia. In parallel, second-generation mitotic drugs have been developed to interfere with proper spindle formation, chromosome segregation, and/or mitotic exit, by targeting PLK1, Aurora kinases, or the APC/C. All these drugs can trigger cell death, the preferred outcome in clinical use. However, they demonstrated limited clinical success in acute leukemias, due to the unfavorable balance between dose-effectiveness and toxicity and the development of some of them has been stopped. The reported adverse effects could be prevented by reducing the inhibitor dose, thus indicating the need to develop effective combinatorial strategies, as observed in the promising combination of the Aurora A inhibitor MLN8237 with chemotherapy. The same success can be achieved by the identification of the right patients, which may benefit of a low inhibitor dose due to a high sensitivity. This approach, based on the concept of gene-to-drug synthetic lethality, has proven highly effective in cancer. Regarding mitotic inhibitors in acute leukemias, preclinical data suggest a very good response of complex karyotype AML to PLK1 inhibition [21] and of cohesion-mutated AML to disruption of APC/C^CDC20^ function [150]. Moreover, most mitotic inhibitors show a SAC-independent and *TP53*-independent activity, which represents a major breakthrough in the field, being *TP53* abnormalities an absolute marker of poor prognosis. Mitotic inhibitors also favor numerical as well as structural chromosomal instability (CIN) and/or tetraploidy-mediated aneuploidy in surviving cells, which are indicative of cancer evolution, and frequently associate with chemoresistance [54]. This downside can provide a therapeutic window for synthetic lethal approaches, in which exposure to one drug greatly sensitizes to treatment with a second agent (drug-to-drug synthetic lethality). This synthetic lethality approach has been already proven in T-ALL. Indeed, a strong synergism using volasertib in combination with MK-5108 on T-ALL cell lines [134]. A preclinical study reported synergistic inhibition of APC/C-dependent proteolysis and mitotic exit by simultaneously disrupting two protein–protein interactions within the APC/C^CDC20^–substrate ternary complex using apcin and proTAME in combination [151].

## Discussion and conclusions

Cancer cells display profound intra- and interline variation following prolonged mitosis. Mitotic slippage is a unique form of cell defense to DNA damage occurring during mitosis and is associated with genetic instability. A better understanding of this process and its molecular players would help define biomarkers that can distinguish between patients who are likely to benefit from a therapeutic regimen including mitotic inhibitors and those who may not benefit due to mitotic slippage. Shedding light on the mechanisms that orchestrate the switch from mitotic slippage to cell death is essential for an informed use of these drugs in the clinical practice. For example, P53 functionality, along with expression levels of CDK1-Cyclin B1 and anti-apoptotic proteins (MCL1, BCL2 or BCL-xL), is critical determinants of cell fate between apoptotic death and mitotic slippage. Indeed, loss of *TP53* and rapid degradation of Cyclin B1 appear to promote mitotic slippage during mitotic arrest. Moreover, it has been hypothesized that mitotic slippage is promoted by the APC/C-dependent degradation of Cyclin B1 in the presence of the active mitotic checkpoint [152]. In this context, overexpression of key molecules of the APC/C may disrupt the balance between mitotic slippage and mitotic death in favor of cell survival. Leukemia-related alterations and patient-specific ones are key determinants of cell fate during mitotic arrest and of response to mitotic inhibitors.

**Fig. 4:**
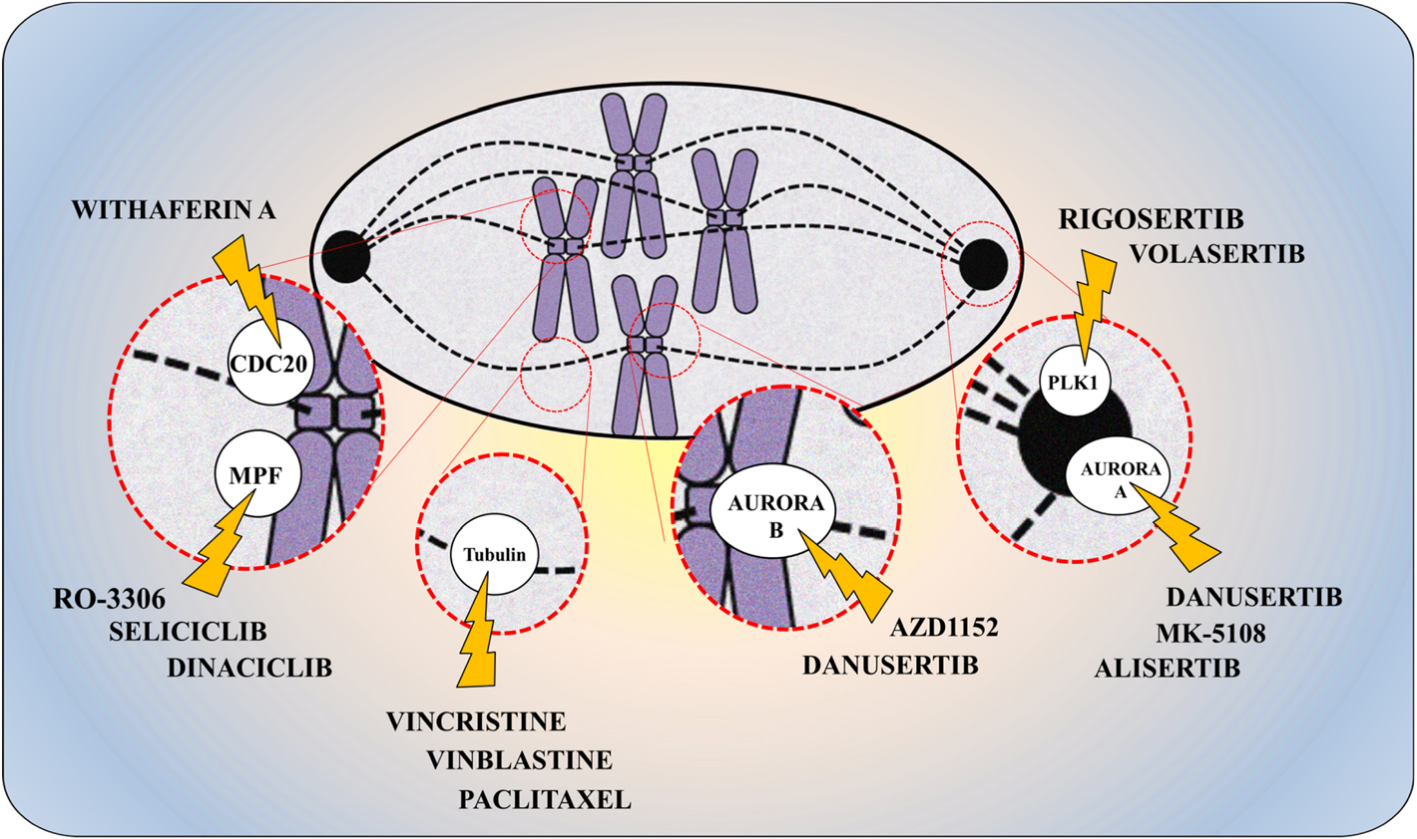
Targeting mitosis using different mitotic inhibitors. Conventional mitotic poisons such as vincristine, vinblastine, or paclitaxel compromise cell proliferation interfering with microtubule assembly and affecting mitosis progression. Innovative selective inhibitors are available to selectively target mitosis in acute leukemia. MPF and CDC20 inhibitors compromise the early phase of mitosis and, in particular, the functionality of SAC complex enhancing mitotic cell death. Aurora kinase and PLK1 inhibitors dysregulate pre-mitotic and mitotic regulation and enhance the efficacy of chemotherapy agents or mitotic poisons

Therefore, the introduction of second-generation mitotic inhibitors could dramatically disrupt the balance between mitotic slippage and mitotic cell death through the selection of the right patients and/or the best combination strategies to target leukemic cells. The genomic and functional characterization of leukemia will help define the therapeutic window to selectively hit cells in a precise mitotic phase, while limiting toxicity, thus facilitating the personalization of acute leukemia therapies (Fig. 4).

## Data Availability

Not applicable

## Abbreviations

ALL: Acute lymphoblastic leukemia
AML: Acute myeloid leukemia
APC: Anaphase promoting complex/cyclosome
CDK: Cyclin-dependent kinase
CR: Complete response
CRi: CR with incomplete blood count recovery
MDS: Myelodysplastic syndromes
MPF: Mitotic promoting factor
MTA: Microtubule targeting agents
OS: Overall survival
OX-1: Oxindole-1
SAC: Spindle assembly checkpoint
VCR: Vincristine
